# Potential Uses, Limitations, and Basic Procedures of Micronuclei and Nuclear Abnormalities in Buccal Cells

**DOI:** 10.1155/2014/956835

**Published:** 2014-02-04

**Authors:** Olivia Torres-Bugarín, María Guadalupe Zavala-Cerna, Arnulfo Nava, Aurelio Flores-García, María Luisa Ramos-Ibarra

**Affiliations:** ^1^Programa Internacional, Facultad de Medicina, Universidad Autónoma de Guadalajara, 45129 Zapopan, JAL, Mexico; ^2^Unidad de Investigación en Epidemiología Clínica, UMAE, HE, CMNO, IMSS, Guadalajara, JAL, Mexico; ^3^Servicio de Medicina Interna, Reumatología e Inmunología Hospital General de Occidente SSJ, Zapopan, JAL, Mexico; ^4^Unidad Académica de Medicina, Universidad Autónoma de Nayarit, 63155 Tepic, NAY, Mexico; ^5^División de Ciencias Veterinarias, Departamento Salud Pública, Centro Universitario de Ciencias Biológicas y Agropecuarias, Universidad de Guadalajara, 45221 Zapopan, JAL, Mexico

## Abstract

The use of biomarkers as tools to evaluate genotoxicity is increasing recently. Methods that have been used previously to evaluate genomic instability are frequently expensive, complicated, and invasive. The micronuclei (MN) and nuclear abnormalities (NA) technique in buccal cells offers a great opportunity to evaluate in a clear and precise way the appearance of genetic damage whether it is present as a consequence of occupational or environmental risk. This technique is reliable, fast, relatively simple, cheap, and minimally invasive and causes no pain. So, it is well accepted by patients; it can also be used to assess the genotoxic effect derived from drug use or as a result of having a chronic disease. Furthermore the beneficial effects derived from changes in life style or taking additional supplements can also be evaluated. In the present paper, we aim to focus on the explanation of MN test and its usefulness as a biomarker; we further give details about procedures to perform and interpret the results of the test and review some factors that could have an influence on the results of the technique.

## 1. Introduction

Biomarkers are biologic parameters that provide information about a physiologic or pathologic state of an individual or a population. In 2001, a consensus panel at the National Institutes of Health defined the term biomarker as a characteristic that is objectively measured and evaluated as an indicator of normal biological processes, pathogenic processes, or pharmacologic responses to a therapeutic intervention or other health care interventions. Biomarkers are potentially useful along the whole spectrum of the disease process. Before diagnosis, markers could be used for screening and risk assessment. During diagnosis, markers can determine staging, grading, and selection of initial therapy. Later, they can be used to monitor therapy, select additional therapy, or monitor recurrent diseases [1]. Moreover, biomarkers detection can be useful tools to evaluate risk factors associated with environmental agent exposure, labor risk, and aging. An ideal biomarker should be safe and specific and must reflect only a subclinical and reversible change; additionally, the collection and analysis of the sample must be simple. The cost of follow-up tests should be relatively low and there should be proven treatment to modify the biomarker; it should be consistent across genders and ethnic groups and must be ethically accepted. Biological markers measured in wild animals can directly contribute to detecting, quantifying, and understanding the significance of exposure to chemicals and biohazards in the environment. These measurements in environmental species may also help assess the potential hazard after human exposure to environmental pollutants, predicting human health risks [2].

## 2. The MN Assay as a Biomarker

The presence of micronuclei (MN) and nuclear abnormalities (NA) are biomarkers broadly used; the detection of MN and NA offers a great opportunity to monitor individuals or populations exposed to mutagenic, genotoxic, or teratogenic events, mainly the evaluation of micronucleogenic cells presence in epithelial tissues.  Table 1 summarizes different studies that have been done mainly in Latin-America in order to demonstrate genotoxicity in association with environmental and job risks. Furthermore, these biomarkers offer additional endpoints for possible evaluation of chromosomal instability and gene amplification (via nuclear buds), cytokinesis arrest due to aneuploidy (via binucleated cells), and different cell death events (e.g., karyorrhectic and pyknotic cells). MN detection can also be used to describe beneficial effects against genotoxicity produced by changes in lifestyle and/or as a consequence of supplements intake [3–6]. It has been described that the choice and the amount of foods and supplements intake have influence on cellular concentrations of micronutrients that are required either as substrates (e.g., 5,10-methylene tetrahydrofolate) or cofactors (e.g., Zn and Mg) in DNA synthesis and repair [7]. Furthermore, it was documented that even small differences in intracellular folic acid concentration *in vitro* induce MN production to the same extent as X-rays of 20 cGy exposure (~20 times the annual safe exposure limit of ionizing radiation) [8]. On the other hand, studies *in vivo* have shown that MN frequency in peripheral blood lymphocytes (PBL) is associated in a significant way with dietary intake and plasma concentrations of folate, vitamin B12, riboflavin, biotin, pantothenate, beta-carotene, vitamin E, retinol, and calcium [9–11]. Further analysis showed that both deficiencies and excesses in micronutrients can be deleterious [12]. About exercise, different studies have reported the effects of it over MN production in PBL, and the results range from a reduction in MN associated with moderate exercise [13] or in individuals that practice exercise frequently [14], null effects [15], to an increase in MN frequency after acute vigorous exercise [16, 17].

The MN test is fast, simple, cheap, minimally invasive, and painless, so, it is well tolerated among patients. Besides, there is no need to perform cell cultures, but, if desired, they can be done [3, 4]. This assay is performed through visualization of cells morphology with a microscope after they are collected, fixed, and stained. MN are formed during the transition metaphase-anaphase of the mitosis and they can appear as complete chromosomes left out usually as a consequence of mitotic apparatus damage (aneuploidogenic effect) or chromosome fragments without centromere (clastogenic damage); in both cases, these genetic materials were unable to be incorporated to daughter cells [18] and they can be differentiated by their size [19] or by centromere presence [20]. Such events can occur in a spontaneous manner; nevertheless, in presence of certain endogenous [21–23] or exogenous factors [3, 24] they seem to be increased. So, MN presence can be used as a biomarker of mutagenic and genotoxic agents influence [25].

The presence of MN can be evaluated in many tissues involving any dividing cell [25], for example, cervix epithelia [26], bladder, esophagus, and bronchial, nasal, and buccal mucosa [3, 4]. Indeed, MN presence has been used as a biomarker of genotoxicity in animals [27–31] and vegetables [32].

Accurately MN presence in cells is observed in epithelial tissue exfoliated cells, derived from the basal layer where cell division takes place and then they migrate towards the surface within 5 to 14 days. In this manner, the epithelial tissue can reflect the damage occurred at this time. Samples can be obtained through a gentle scrape of the tissue; then they are placed on a polished slide and with another slide a smear is done; after this, cells are air-dried and fixed with 80% ethanol or 80% methanol (we suggest the use of ethanol over methanol because the latter can be toxic during evaporation) before staining and finally analyze cells from 500 to 4000 with a microscope and register the number of MN found [5].

Repeatedly, the oral cavity has been proposed as a mirror that reflects an individual's health, since oral mucosa often reflects disease changes; furthermore, it is the first contact with many pollutants like tobacco or alcohol and its affection can also be indicative of a systemic condition or side effects due to chemotherapy or radiotherapy administration [5, 33].

Oral mucosa provides an easy access to sample collection with a technique that is minimally invasive and painless; for these reasons, it is well tolerated among patients and shows the first candidate tissue that serves to evaluate cytotoxic and genotoxic effects. Additionally, oral mucosa is the first line of contact with different hazardous agents; it provides the first barrier against potential carcinogens and is therefore susceptible to damage by these agents before reflecting a systemic condition. This epithelium has a unique proliferative response which allows cellular population to maintain a constant rate of cell divisions; nevertheless, this characteristic makes cells prone to DNA damage, a finding that is relevant since it is estimated that 90% of all cancers are derived from epithelial cells [5].

Oral mucosa cells are useful for determining exposure to compounds not only because they are the first line of encounter with several environmental factors like tobacco and alcohol, but also since several systemic conditions and treatments limit the proliferation rate of epithelial cells [33]. Furthermore, it is important to outline the fact that nearly 60% of the oral mucosa surface is stratified nonkeratinized epithelia, which allows cells in the most superficial layer to maintain their nuclei well defined and almost intact, a characteristic that favors colorant absorption and eases observation and proper identification of nuclei cells morphologic characteristics with the use of a microscope. Moreover, keratinocytes are big cells with abundant cytoplasm [33] and they can be studied without the need of a cell culture, which makes this test both simple and cheap. For all of these reasons, cells derived from oral mucosa can be used to monitor early genotoxic events caused by ingestion or inhalation of carcinogens [34]; the capability for test performance also makes it ideal for the study of whole populations with increased risk or susceptible to cytotoxic damage by means of MN and NA detection [35]. The MN assay can also be used for epidemiologic studies with life style impact, occupational exposure, nutrition, chronic disease evolution, cancer, aging, and drug effects, among others [3, 24, 36, 37].

## 3. Nuclear Abnormalities in Buccal Cells

Besides MN presence, Tolbert and cols in 1991 [38] described other NA as phenomena that can occur not only during physiologic cellular differentiation but also during death cell with DNA damage. Since most changes in a neoplastic cell are being produced in the nuclei, with modifications being changed in size, density, and chromatin distribution, such abnormalities can be distinctive between normal cells and affected ones. NA involve condensed chromatic (CC), karyorrhexis (KR), pyknotic nuclei (PN), karyolysis (KL), nuclear buds (NBUDs), and presence of cells with two nuclei called binucleated (BN). The mechanisms through which each of these abnormalities is produced or its biological meaning is still unknown. Nevertheless, under pathological conditions (obesity, rheumatoid arthritis, systemic lupus erythematosus, cancer, lymphoma, leukemia, multiple myeloma, thrombocytopenic purpura, and others) or after exposition to tobacco, alcohol, and drugs, a higher frequency in NA can be seen [37]. Although the exact mechanism for NA production has not been conclusive, some authors have referred hypothesis about possible mechanisms of NA formation or their biological meaning. Precisely, Raj and cols described MN and NA presence with an effect dose response after radiotherapy in patients with oral squamous cell carcinoma, suggesting the use of these biomarkers to assess radiosensitivity [39]. NA are also present in ageing process as described by Thomas and cols, where MN, NBUDs, and BN are increased in subjects aged between 64 and 75 years and also in Down syndrome patients [40]. NA presence has been considered a biomarker for DNA damage (MN and NBUDs), cytokinesis defects (BN), cell death evidence (CC, KR, PN, y KL), different stages of necrosis indicators (PN, CC, KR, and KL), and identifying the response to cell damage (PN and CC). Some researchers consider lymphocyte NBUDs as indicators of genotoxicity. Nevertheless, in exfoliated cells, remains controversial since in different process of health and disease micronucleated cells can appear in an increased manner and NBUDs presence is not correlated with MN number [41]. Their structure is suggestive of a budding process involved in the elimination of excess nuclear material such as unresolved DNA repair complexes or amplified DNA following its segregation to the periphery of the nucleus [42]. For NA identification, we suggest the use of classification criteria proposed by Tolbert et al. (1992) [6, 35, 40].

## 4. Protocol for MN and NA Detection in Buccal Cells *In Vivo*

### 4.1. Ethical Issues

Previous to any procedure, a written informed consent must be obtained in accordance to Helsinki's Declaration, World Medical Association, and institutional as well as governmental regulations.

### 4.2. Patient Information

It is important to obtain relevant information from the patient, like gender, age, weight, height, lifestyle, general health, intake of coffee, smoking habits, use of drugs, treatments, and last visit to the dentist (anesthetic use, tooth extraction, etc.) in order to determine possible causes other than a pathology for the presence of elevated MN or NA.

### 4.3. Control Selection

Healthy subjects with clinically normal oral mucosa and good dental hygiene. It is ideal that the subject does not smoke or drinks coffee. The subject should not be obese and should not be exposed to pesticides or any other substance known to be genotoxic. Furthermore, the use of antioxidants, vitamins, and supplements in general should be considered, as well as turnover rate of oral cells.

### 4.4. Sample Collection

For genotoxicity evaluation in cells, it is convenient that each participant had a mouth wash with water before sample collection in order to remove any food or artifacts that may interfere with the analysis. The sample will be collected with a gentle swab of the oral mucosa of the right and left cheeks with a polished slide then the samples are spread directly in two separate slides. It is convenient to make two smears of each cheek in case the first one can not be analyzed. It is important to consider that collection of the sample should be done with the same strength and skill to obtain a single layer of epithelia; in other words, sampling method should be kept constant since, repeated and vigorous sampling may lead to collection of cells from the less differentiated basal layer.

### 4.5. Sample Codification

It is desirable that the code used to classify the slides is consecutive to avoid the use of terms that can lead to identification of the sample by the sample reader. It is necessary to keep a clear and precise registry of each patient and their codes recorded in different files to avoid losing information.

### 4.6. Fixation

Smears are air-dried and fixed with 80% ethanol for 48 hours before they can be stained.

### 4.7. Stain

The colorant used should be basic and with high affinity to DNA in order to obtain a contrast so one can be able to differentiate artifacts. We suggest the use of acridine orange which is specific stain for DNA and therefore allows differentiation with RNA; other stains include Feulgen, Shiff, Papanicolaou, orcein, or hematoxylin and eosin; all of them are acid-basic stains and they can produce a contrast between the cytoplasm and the nucleus. It is recommended to avoid stains that can't differentiate between cells and artifacts (Giemsa, May-Grünwald-Giemsa) since they can favor false positive readings and micronuclei formation in epithelial cells may be overestimated when non-DNA-specific stains are used [3, 43–45].

#### 4.7.1. Acridine Orange

Cells that are undergoing mitosis with disperse chromatin (abundant DNA) are stained in green and cells during interphase (condensed chromatin and increased number of RNA and proteins) are stained in orange. 

(*i) Stain Preparation Procedure.* You will need two separate containers for slides; add 1/2 Lt of phosphate buffer previously prepared (save 10 mL in a sterile syringe) to each container. Then, add to one of the containers 0.05 gr of acridine orange dust and gently agitate; once it is homogenized, insert the slides for 5–7 min; for better results, try to gently agitate the solution occasionally with the slides until time completion. Next, discard the colorant solution and leave the slides to dry off in the container and proceed to wash the slides in the second container with phosphate buffer solution. Once the staining of all slides is completed, smears should be stored in a dark box at room temperature until reading under the microscope. As a suggestion, try coloring one slide and see under the fluorescence microscope if the stain is ideal; otherwise, you can add more powder or decrease the amount for the rest of the slides staining [46].

(*ii) Phosphate Buffer.*Prepare a mixture of monobasic dihydrogen phosphate (11.49 gr) and dibasic monohydrogen phosphate (2.16 gr). By varying the amount of each salt, a range of buffers can be prepared that buffer well between pH 5.8 and pH 8.0. Phosphates have a very high buffering capacity and are highly soluble in water. Add 1 Lt of distilled water and mix until the powder is entirely dissolved. The solution can be stored at 4°C until used, preferably no more than 30 days. 

(*iii) Smear Preparation for Observation under the Microscope*. Wear gloves and lab coat (the colorant is mutagenic), add a drop of phosphate buffer over the simple (from the 10 mL syringe reserved earlier), place the cover slip avoiding bubble formation, surround it with a clean lint and press gently, take it under the microscope under the 100x lens, add a drop of immersion oil, and observe. It is convenient to finish the reading once a slide is started, otherwise it will dry out or can be contaminated and the intensity of color might decrease favoring false negative readings.

(*iv) Advantages and Disadvantages of the Acridine Orange Use*. It is important to point out that the use of this colorant decreases the time for analysis in a 50%, from the stand point of view that an expert will take up to 1 hour to analyze the sample with other colorants. The use of the colorant should be done with extreme caution since it is highly genotoxic and mutagenic. Due to its nature as a base analogue, it can cause frame shift mutations. Acridine orange is more expensive than other colorants, but the amount needed for each slide is less than that needed for other colorants. Another consideration about costs is the fact that a fluorescence microscope needs proper maintenance programs for it, including the mercury lamp replacement after 100–120 hours of use. New microscopes include a led lamp. Although it is more expensive, the replacement is no needed for this microscope. Advantages include the fact that precision in identification of MN is very high. 

#### 4.7.2. Orcein

After immersion in 1 N HCl at 60°C for 8 min, make a second immersion of the slides in orcein reagent at 40°C for 20 min (or immerse only in orcein for 2 hours) then rinse with ethanol and then distilled water. Use fast green contrast for 30 seconds and rinse with distilled water. After this procedure, the nucleus is stained in pink or orange and the cytoplasm in blue [22, 24].

*Orcein and Fast Green Preparation*. Heat 30 mL of acetic acid and dissolve 1 gr of orcein in it then wait for it to cool down and add 20 mL of distilled water. Fast green; Stock: Dilute 0.2 gr of fast green in 100 mL of water and add 0.2 mL de of glacial acetic acid. Work solution: take 10 mL from the stock solution and add 50 mL of distilled water.

#### 4.7.3. Shiff Reagent

This colorant allows the observation of the cytoplasm in a characteristic red due to the presence of glycogen; mucin and the nuclei are stained in blue. Once the slides are fixated, immerse them in 0.5% periodic acid for 15 minutes, wash with tap water for 5 minutes and then add the Schiff reagent for 10 to 15 minutes; after this time, make a second wash with tap water for another 5 minutes. Then, expose the slides to hematoxylin for 3 to 5 minutes and finally wash them for another 5 minutes in tap water, and preserve the slides in a box until read.

*Shiff Reagent Colorant Preparation*. Dissolve 1 gr of basic fuchsine in 200 mL of distilled water and heat to 80°C, let it cool down, and add 2 gr of sodium or potassium metabisulfite, mix, and incorporate 10 mL of concentrated hydrochloric acid or 2.5 mL of 1 N. Before use mix and heat to boil and add 0.5–1 gr of activated carbon; let it settle down for 24 hours and filtrate, until the liquid is clear. For storage, use an amber colored jar maintained at 4°C.

#### 4.7.4. Modified Feulgen

Using Feulgen stain with the following modifications, perform a hydrolysis by immersing the slides in 1 N HCl at room temperature for 1 min, then in 1 N HCl at 60°C for 10 min, and finally in 1 N HCl at room temperature, for 1 min; after this, immerse the slides in Schiff's reagent for 90 min and rinse with distilled water, then contrast with Fast Green for 30 s and rinse again with distilled water. During the observation of the slides, the nucleus must be stained in pink or orange and the cytoplasm in blue [36].

#### 4.7.5. Hematoxilin-Eosin

This technique requires two colorants. First, samples are exposed to hematoxilin, basic dye that binds to any substance that contains acid groups such as the phosphate groups in the DNA structure and also binds to nuclear proteins with negative charge. Then, samples can be exposed to eosin, a weak acid colorant that stains basic structures. The basophil structures like nuclei are stained in color blue with hematoxilin, while eosin stains in pink acidophil structures like collagen fibers. Procedure: immerse the samples in an alcohol series (100°, 95° y 70°), wash with tap water to eliminate alcohol excess, then immerse the slides in hematoxilin for 10 minutes. Make another washing with tap water and rapidly immerse in acid alcohol. Wash again and immerse for 30 s in eosin, go through the alcohol series again, this time in crescent order (70°, 95° y 100°), and store in boxes until use. 

*Advantages and Disadvantages of Orcein, Shiff Reagent, and Hematoxiline-Eosin Use*. In general, these techniques are elaborated procedures and under precipitation of the colorants difficulties during observation can be presented, which could facilitate a false positive result during MN and NA assessment. Alternatively, if enough precautions are taken, these are very reliable methods. The Feulgen method is very specific for DNA identification; RNA is not stained due to a weak acid hydrolysis with hydrogen chloride (HCl) and purine groups of the DNA can be separated. In this manner, desoxyribose ring can detach and form an aldehyde group that reacts with Shiff reagent resulting in a dark pink or red color. On the other hand, hematoxilin is a compound obtained from leguminous *Haematoxylum campechianum*, known as “Palo de Campeche.” It is a natural compound that, once oxidized constitutes a purple substance denominated hematin. It is used in histological reactions to dye anionic components (acids) from tissues, in violet color. Nuclei are intensely dyed, since they contain nucleic acids which are abundant in acid radicals. Furthermore, hematoxilin is neutral, but it is often a denominated basic colorant since its chromogenic compound resides in the cationic (basic) complex of it.

### 4.8. Slides Analysis

The person designated to analyze the samples must not be aware of sample codification. Under the microscope with the immersion objective, at least a count of 2000 cells per patient must be done. According to Ceppi et al. [47], it is recommended to analyze from 3000 to 4000 cell; nevertheless, in our experience the analysis of 2000 cells is both reliable and cost-effective. Once this is achieved, proceed to register the number of micronucleated (MN) cells and describe the presence of nuclear abnormalities (NA). It is suggested to follow the classification criteria established by Tolbert et al., 1992 [35]. It is recommended that the analysis is done by the same observer, in the case that another observer should be added to the analysis, and then a standardization of the analysis should be done, through repeated readings of the same sample and unification of criteria. At the end of the standardization process, a statistical analysis should be done between the results of the two observers and establish the intervariability coefficient.

### 4.9. Classification Criteria

#### 4.9.1. Normal Cells (NC), Figure 1(a)

The nucleus is uniformly stained, oval- or circle-shaped, and smaller than the cytoplasm. Basal cells are distinguished because they are bigger. There is absence of any other structure besides from the nucleus that contains DNA; these cells are considered as completely differentiated cells.

#### 4.9.2. Micronucleated Cell (MN), Figure 1(b)

Characterized by the presence of a main nucleus and smaller structures denominated micronuclei (MN). One MN has circle or oval shape and its length is between 1/3 and 1/16 of the main nucleus, the intensity of the stain, texture and plane is equal in both structures. MN characteristics are rounded smooth perimeter suggestive of a membrane, less than a third the diameter of the associated nucleus, but large enough to discern shape and color; staining intensity similar to that of the nucleus; similar texture to that of nucleus; same focal plane as nucleus; and absence of overlap with, or bridge to, the nucleus.

#### 4.9.3. Binucleated Cells (BN), Figure 1(c)

They are cells with two main nuclei, and usually both nuclei are in close proximity or even in contact, both with similar shape, and stain to a normal nucleus. Its formation seems to be related to interference during the procedures at the end of cell division.

#### 4.9.4. Karyorrhexis (KR), Figure 1(d)

The nucleus is characterized by chromatin aggregation, visualized as a pattern denominated nuclear spotted, indicative of nuclear fragmentation which will lead to nuclear disintegration. These cells might be undergoing an advanced phase of apoptosis, but this is still unclear.

#### 4.9.5. Condensed Chromatin (CC), Figure 2(a)

These nuclei are intensively stained, with chromatin aggregates exhibiting a pattern denominated nuclear spotted or nuclear striated. It is evident that chromatin is aggregated in some nucleus regions, while other areas lack the presence of chromatin. When the condensation is extended, it appears like a fragmented nucleus; these just like kariorrhexis cells end up with nuclear fragmentation, which leads to eventual disintegration of the nucleus. Condensed chromatin might represent initial stages during apoptosis, but this is still not conclusive.

#### 4.9.6. Pyknotic Nuclei (PN), Figure 2(b)

The nucleus appears small in size, with high density of nuclear material which is uniformly distributed, but highly stained. Nucleus diameter is approximately 1/3 of normal nucleus. Biological meaning of PN is still unknown, but we think that they represent death cells. Its presence is correlated with differentiation and maturation of epithelial cells.

#### 4.9.7. Karyolysis (KL), Figure 2(c)

The nucleus has complete lack of DNA; they probably represent an advanced stage of cellular death. A positive correlation between pyknotic cells and karyolysis has been described and it suggests that cells with karyolysis are derived from condensated chromatin cells or indirectly from PN cells.

#### 4.9.8. Nuclear Buds (NBUDs), Figures 2(c) and 3

**Figure 3:**
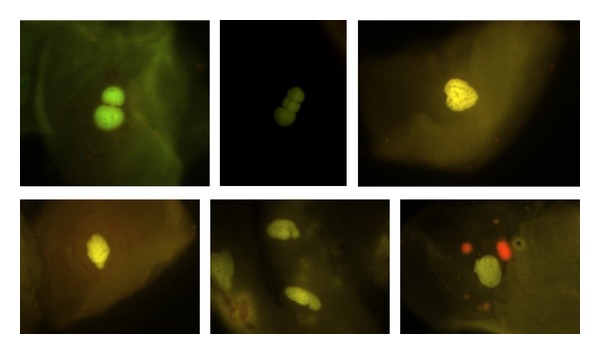
Typical and atypical nuclear buds (NBUDs). Photomicrographs stained with acridine orange viewed at 1000 magnification under fluorescence with an IVFL filter (450–490 nm). Binocular Microscope Carl Zeiss (Axiostar Plus).

The nucleus presents a strong constriction in one extreme, suggestive of nuclear elimination material process by budding formation. The lobule has similar characteristics in morphology to the nucleus but its size is 1/3 to 1/4 of the main nucleus. Tolbert et al., 1991, defined them as “Broken eggs.” Later in 1998 Bhattathiri et al. described this same phenomenon in exfoliated cells as “Nuclear buds” defined as genetic material budding formation in close proximity to the nucleus without a clear separation; lately, Cerqueira in 2004 described Broken eggs and nuclear buds as totally different events. Nevertheless for scoring purposes NBUDs and broken eggs are classified together into a single NBUDs category. In some cases, NBUDs may be even larger, sometimes almost up to the size of the main nucleus. Because these larger nuclear buds, originally designated by Domínguez et al. [48] as “broken egg,” are of uncertain origin, they, for practical reasons and because of their similar appearance, included in the NBUDs category. According to Bolognesi et al. (2013), cells with NBUDs have the following characteristics [42]: (1) the main nucleus has a sharp constriction forming a bud of nuclear material, (2) NBUDs are attached to the main nucleus by a narrow or wide nucleoplasmic bridge, (3) NBUDs and their associated nucleoplasmic bridges have similar staining intensity as the main nucleus, and (4) they usually have a diameter that is 1/3rd–1/16th of that of the main nucleus but in some rarer case (broken eggs) could be even greater and almost up to the same size as the main nucleus. Examples of cells with typical NBUDs are shown in Figure 3(a). Examples of cells with “broken egg” NBUDs are shown in Figures 3(b), 3(c), and 3(d).

## 5. Conclusion

The MN assay in buccal cells has been widely applied worldwide and its use has been increasing in the last decade. Conditions that facilitate its use include the fact that it can be easily done and has a minimally invasive sampling procedure. The most common application of the MN assay in buccal cells concerns occupational and environmental exposure to genotoxic agents, in this field, a diversity of biomarkers exist. In toxicology, is recognized the fact that more than one test in necessary in order to demonstrate a causal effect of a determinant pollutant. MN and NA test is a versatile biomarker that is reliable to measure genotoxic, mutagenic, and teratogenic events. It can also provide valuable information on the stage of progression of some degenerative diseases. However, the role of the MN and NA assay and its applicability in human populations needs to be established and to be more clearly defined. Currently, the HUMN database offers the possibility to unify criteria and standardize different techniques. A correct study design considering the best sampling time relative to exposure period is needed mainly in the application of the assay for discontinuous exposures; improvement in reproducibility and sensitivity of the test in risk prevention and management of workers subject to occupational genotoxic exposures; may be achieved by using a single DNA specific staining method for slide preparation and a clear definition of the scoring criteria. Furthermore, frequency of MN and other NA in buccal cells from healthy subjects and the role of technical and biological confounding factors relevant for its variability need to be clearly defined in order to improve the sensitivity and potential specificity of the assay. The biological meaning of other nuclear alterations as biomarkers of different toxic or genotoxic events or as predictive parameters for cancer or other degenerative diseases needs to be further explored.

The more detailed description of the scoring criteria and the photomicrograph gallery provided in this paper is valuable for regularization in sampling, staining, and scoring procedures in the realization of the MN and NA test.

## Figures and Tables

**Figure 1:**
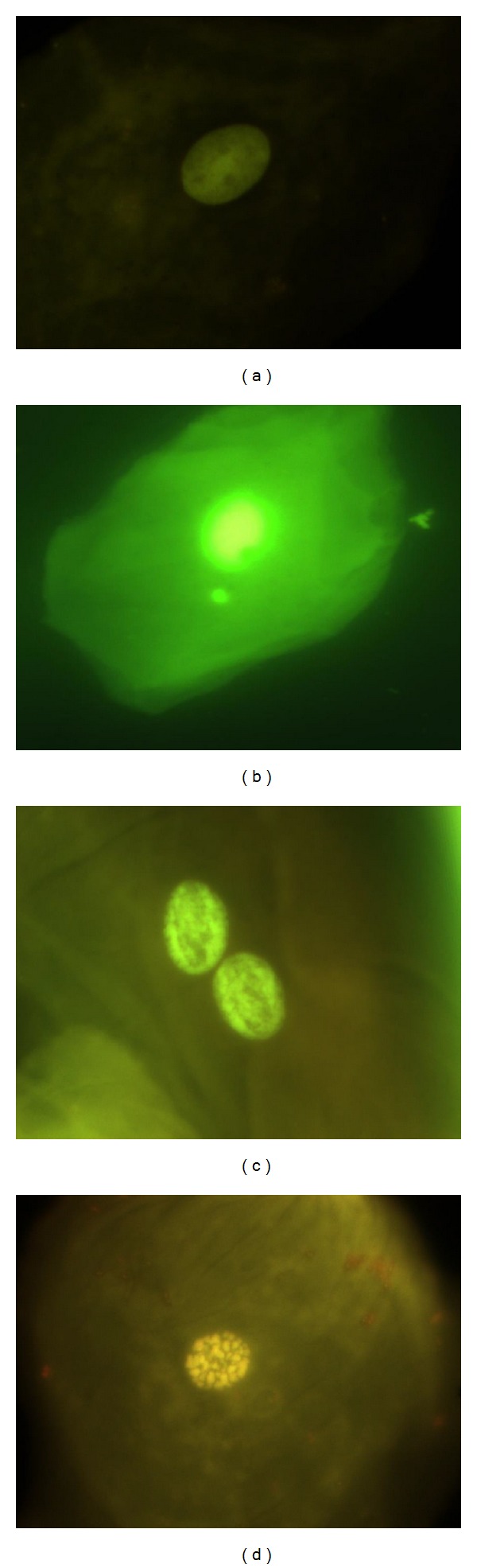
(a) Cells with normal nucleus. (b) Micronucleated cell (MN), (c) binucleated cells (BN), and (d) karyorrhexis (KR). Photomicrographs stained with acridine orange viewed at 1000 magnification under fluorescence with an IVFL filter (450–490 nm). Binocular Microscope Carl Zeiss (Axiostar Plus).

**Figure 2:**
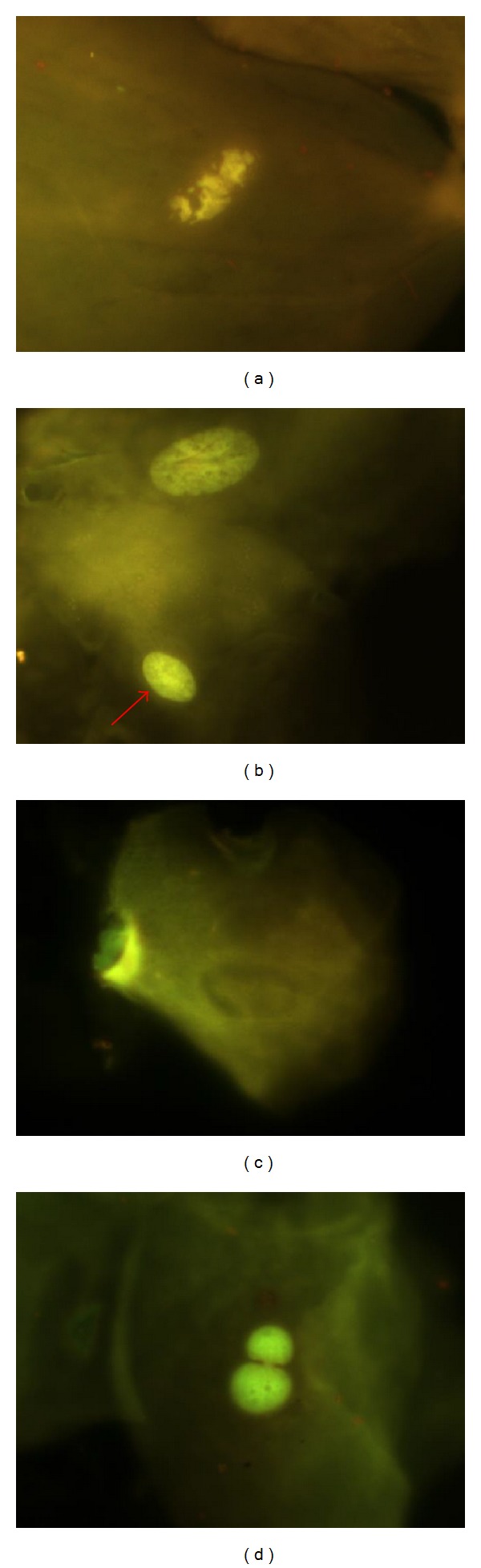
(a) Condensed chromatin (CC), (b) pyknotic nuclei, arrow (PN), (c) karyolysis (KL), and (d) typical nuclear buds (NBUDs). Photomicrographs stained with acridine orange viewed at 1000 magnification under fluorescence with an IVFL filter (450–490 nm). Binocular Microscope Carl Zeiss (Axiostar Plus).

**Table 1:** The use of micronuclei and nuclear abnormalities as markers for labor and environmental risk.

Labor/environmental risk	Age (years)	MN	CC	KR	PN	KL	NBUDs	BNC	CA stained	Country reference
Banana harvester (pesticide)									1000 (ND)	Costa Rica, 2004 [49]
Exposed (*n* = 40)	16–50	0.03	0.003	0.04*	0.03	0.08	0.02	0.04		
Nonexposed (*n* = 44)	33.2	0.03	0.003	0.06	0.04	0.1	0.02	0.04		

Floriculture (pesticide)									1000 (1)	Mexico, 2000 [50]
Exposed (*n* = 30)	18–63	10.01 ± 0.03*	19 ± 0.0	2 ± 0.0*	4 ± 0.0*	8 ± 0.0*	0.7 ± 0.0	25 ± 0.0		
Nonexposed (*n* = 30)		0.3 ± 0.02	19 ± 0.0	0.5 ± 0.0	2 ± 0.0	3 ± 0.0	0.1 ± 0.0	16 ± 0.0		

Pesticide production									2000 (6)	India, 2006 [51]
Exposed (*n* = 54)	35.1 ± 5.2	1.2 ± 0.7*	ND	ND	ND	ND	ND	ND		
Nonexposed (*n* = 54)	33.4 ± 5.5	0.3 ± 0.2								

Nurses (cytostatic drugs)									1000 (2)	Cuba, 2004 [48]
Exposed	39	7.3*	7.0*	ND	5.09*	9.1*	ND	8.09*		
Nonexposed		4.5	1.5		4.1	1.8		4.6		

Cone beam computed tomography									2000 (1)	Brazil, 2010 [52]
Before exposure (*n* = 19)	26.8 ± 5	0.04 ± 0.05	ND	7.4 ± 46 (KR, PN, KL)			ND	ND		
After exposure (*n* = 19)		0.05 ± 0.06		17.8 ± 5.4* (KR, PN, KL)						

Chemical products									1000 (2)	Cuba, 2005 [53]
Nonexposed (*n* = 20)	36.2 ± 5.9	5.6	4.2		7.4	4.5	ND	9.2		
Carbon dioxide (*n* = 20)	52.2 ± 7.0	5.4	6.5*		5.5	5.6		5.2		
Ammonia (*n* = 15)	35.7 ± 5.7	8.1*	8.3*		7.7	7.6		10.0*		
Welding vapor (Fe, Zn, Ni, and Cr) (*n* = 11)	40.7 ± 8.7	8.6*	9.5	ND	8.5	10.0*		13.2*		

Welder (Fe, Zn, Ni, and Cr vapor)									1000 (2)	Colombia, 2011 [54]
Exposed (*n* = 36)	ND	0.5 ± 0.1*	ND	ND	ND	ND	0.2 ± 0.04*	13.7 ± 0.7*		
Nonexposed (*n* = 25)		0.2 ± 0.07					0.05 ± 0.02	5.6 ± 0.3		

Children with metal crowns (nickel)									1000 (2)	Mexico, 2013 [55]
Day 1 (*n* = 37)	4–11	4.6 ± 0.15	ND	ND	ND	ND	ND	ND		
Day 45 (*n* = 37)	6.2 ± 1.7	6.7 ± 0.16*								

Lead battery factory workers (smokers)									1000 (2)	India, 2011 [56]
Exposed (*n* = 62)	38.0 + 7.0	10.1*	ND	18.8*		46.0	6.8	ND		
Nonexposed (*n* = 60)	42.2 + 8.0	3.6 ± 0.1		8.6 ± 0.4		32.4 ± 0.6	3.7 ± 0.4			

Chrome plating workers (hexavalent Cr)									1500 (3)	India, 2011 [57]
Smokers exposed (*n* = 24)	43.4 ± 3.7	4.5 ± 1.1*	ND	36.2 ± 5.9	5.1 ± 0.4	128.9 ± 8.4	7.1 ± 1.5	7.2 ± 0.8		
Exposed (*n* = 20)	37.6 ± 2.9	3.1 ± 1.1*		52.2 ± 7.0	3.6 ± 0.3	87 ± 4.2	5.0 ± 0.7	6.9 ± 0.9		
Smokers nonexposed (*n* = 21)	42.5 ± 2.2	1.4 ± 0.4		9.1 ± 2.9	2.6 ± 0.2	39.2 ± 2.4	2.5 ± 1.4	4.7 ± 0.8		
Nonexposed (*n* = 19)	40.2 ± 3.7	1.0 ± 0.2		6.0 ± 1.56	1.8 ± 0.7	26.3 ± 2.5	3.2 ± 1.5	3.4 ± 0.9		

Urban soil (arsenic and lead)									1000 (ND)	Mexico, 2013 [58]
Exposed (*n* = 98)	4–10	2.2 ± 1.6*	ND	ND	ND	ND	ND	ND		
Nonexposed (*n* = 42)		1.06 ± 0.74								

Glass workers (arsenic)	18–34	0.4 ± 0.1	ND	ND	ND	ND	ND	ND	2000 (1)	India, 2006 [59]
Exposed (*n* = 200)	35–68	1.1 ± 0.1*								
Nonexposed (*n* = 165)	18–34	0.1 ± 0.04								
	35–68	0.2 ± 0.05								

Outdoor painters									3000 (1)	Mexico, 2000 [60]
Exposed (*n* = 25)	25–60	1.1 ± 0.02*	ND	ND	ND	ND	ND	ND		
Nonexposed (*n* = 25)		0.3 ± 0.01								

Commercial painters									3000 (1)	India, 2010 [61]
Exposed (*n* = 30)	20–50	17.7 ± 3.2	ND	23.8 ± 4.2*	ND	21.3 ± 4.5*	10.4 ± 3.4*	25.7 ± 4.5*		
Nonexposed (*n* = 30)		2.5 ± 1.3		2.7 ± 0.6		2.7 ± 0.8	0.6 ± 0.4	2.3 ± 0.9		

Shoe workers (toluene and others)									1000 (1)	Mexico, 2009 [62]
Exposed (*n* = 34)	18–65	0.1^∗♦^	5.0^♦^	0.0^♦^	1.0^♦^	0.2^♦^	0.1^♦^	0.3^♦^		
Nonexposed (*n* = 34)		0.0^♦^	6.1^♦^	0.0^♦^	0.7^♦^	0.0^♦^	0.0^♦^	0.2^♦^		

Tannery workers									2000 (2)	India, 2011 [63]
Exposed (*n* = 84)	37.4 ± 8.6	7.1 ± 1.1*	ND	ND	ND	ND	ND	ND		
Nonexposed (*n* = 52)	37.6 ± 8.4	4.0 ± 1.6								

Power plant processing poultry litter workers									2000 (3)	Austria, 2012 [64]
Exposed (*n* = 21)	41.2 ± 9.4	No difference	No difference	*	*	No difference	No difference	No difference		
Nonexposed (*n* = 25)	41.1 ± 8.1									

Formaldehyde									1000 (1)	Portugal, 2010 [65]
Pathology laboratory (*n* = 50)	33.8 ± 8.2	0.6 ± 1.7	ND	ND	ND	ND	ND	ND		
Formaldehyde-based resins production factory (*n* = 30)		1.2 ± 1.5*								
Nonexposed (*n* = 85)	35.7 ± 9.4	0.1 ± 0.4								

Hairdressers									2000 (3)	Brazil, 2010 [66]
Exposed (*n* = 50)	37.4 ± 2.0	2.0 ± 3.6*	ND	ND	ND	ND	9.0 ± 3.8	8.5 ± 5.0		
Nonexposed (*n* = 50)	37.0 ± 12.0	0.3 ± 1.0					5.9 ± 2.6	5.2 ± 4.7		

Thermoelectric plant workers									1000 (1)	Portugal, 2012 [67]
Exposed (*n* = 44)	36.2 ± 9.6	1.8 ± 1.6*	ND	82.4‰ (karyorrhexis, pyknosis, and karyolysis)*			ND	ND		
Office nonexposed (*n* = 47)	42.1 ± 7.6	0.2 ± 0.4		58.3‰ (karyorrhexis, pyknosis, and karyolysis)							

Calcite factory workers									1000 (2)	Turkey, 2010 [68]
Smokers + CaCO_3 _(*n* = 25)	30.7 ± 0.7	11.4 ± 1.1		3.0 ± 0.2		1.9 ± 0.4	15.1 ± 1.5	11.2 ± 0.8		
Nonsmokers + CaCO_3_ (*n* = 25)		4.6 ± 1.1	ND	2.2 ± 0.2	ND	3.1 ± 0.4	4.0 ± 1.5	6.9 ± 0.8		
Smokers Nonexposed (*n* = 25)	30.6 ± 1.2	25.5 ± 1.1		1.1 ± 0.2		1.2 ± 0.4	3.5 ± 1.5	4.7 ± 0.8		
Nonsmokers (*n* = 25)		15.1 ± 1.1		1.0 ± 0.2		1.3 ± 0.4	5.2 ± 1.5	3.4 ± 0.8		

Fisher folks/mine tailings									1000 (1)	Philippines, 2010 [69]
Exposed (*n* = 47)	8–70	7.7 ± 2.9*	ND	ND	ND	ND	ND	ND		
Nonexposed (*n* = 21)		2.2 ± 1.5								

Mechanic and car painters									3000 (1)	Brazil, 2000 [70]
Exposed (*n* = 60)	15–50	8.2 ± 4.3*	ND	ND	ND	ND	ND	ND		
Nonexposed (*n* = 80)		2.1 ± 1.6								

Firefighter			ND					ND	1000 (3)	India, 2005 [71]
Exposed (*n* = 47)	43.2	3.9 ± 0.1*		24.1 ± 1.4*	2.8 ± 0.4*	152.6 ± 10.1*	5.6 ± 0.4*			
Nonexposed/office (*n* = 40)	44	1.2 ± 0.01		6.45 ± 0.76	0.62 ± 0.13	21.5 ± 2.2	1.7 ± 0.2			

Fire breathers									1000 (4)	Mexico, 1998 [36]
Exposed (*n* = 5)		0.8 ± 0.7	2.4 ± 2.9*	0.4 ± 0.6*	0.7 ± 0.8*		0.8 ± 0.9	4.2 ± 2.3*		
Exposed + smokers (*n* = 3)	28–54	0.7 ± 0.5	2.9 ± 1.1	4.4 ± 0.8	1.3 ± 0.4	0	0.4 ± 0.5	5.8 ± 1.1		
Nonexposed (*n* = 13)		1.1 ± 0.9	1.3 ± 1.6	0.1 ± 0.1	0.7 ± 1.4		0.7 ± 0.8	2.7 ± 1.7		

Fuel dispenser									1000 (1)	Turkey, 2003 [72]
Smokers (*n* = 25)		1.6 ± 0.08*		0.5 ± 0.09		0.4 ± 0.1		2.3 ± 0.4		
Nonsmokers (*n* = 25)		1.1 ± 0.06		0.3 ± 0.09		0.3 ± 0.0		1.7 ± 0.05		
Nonexposed			ND		ND		ND			
Smokers (*n* = 25)		0.6 ± 0.03		1.8 ± 0.1		1.7 ± 0.1		0.8 ± 0.0		
Nonsmokers (*n* = 25)		0.2 ± 0.02		1.3 ± 0.1		1.5 ± 0.1		0.6 ± 0.0		

Fuel dispenser									1000 (2)	India, 2010 [73]
Smokers (*n* = 56)	17–35	9.8 ± 2.0*	ND	19.8 ± 03*		41.2 ± 0.5	ND	7.8 ± 1.0*		
Nonsmokers (*n* = 64)		7.1 ± 3.1		7.8 ± 0.5		32.8 ± 0.2		4.8 ± 1.0		
Nonexposed										
Smokers (*n* = 30)		2.9 ± 0.01		9.6 ± 1.1		29.4 ± 0.5		3.4 ± 0.4		
Nonsmokers (*n* = 75)		2.1 ± 0.02		7.6 ± 0.7		24.4 ± 1.1		2.8 ± 0.1		

Fuel dispenser (benzene)									1000 (5)	India, 2011 [74]
(*n* = 200)			ND		ND					
1.500 *μ*g m^−3^	37.5 ± 6.3	5.1 ± 0.3*		1.1 ± 0.3*		2.2 ± 0.1*	1.0 ± 0.0	3.0 ± 0.01*		
1.100 *μ*g m^−3^	34.8 ± 6.2	2.7 ± 0.4		0.03 ± 0.1		0	0	1.04 ± 0.2		

MN: micronuclei; CC: condensed chromatin; KR: karyorrhexis, PN: pyknosis; KL: karyolysis; NBUDs: nuclear buds; BN: binucleated cells; AC: analyzed cells; ND: no data. All values are represented as mean ± SD, except for values marked with ^♦^represent median. *Statistical difference when compared with reference group.
